# Chronic spontaneous urticaria treatments and purinergic signaling: a therapeutic possibility

**DOI:** 10.1007/s00109-026-02670-0

**Published:** 2026-03-31

**Authors:** Michelli Fontana, Júlia Leão Batista Simões, Geórgia de Carvalho Braga, Mauricio Fontana, João Carlos Menta Filho, Margarete Dulce Bagatini

**Affiliations:** 1 Medical School, Federal University of Fronteira Sul, Chapecó, SC Brazil; 2 Medical School, Catholic University of Pelotas, Pelotas, RS Brazil; 3 Graduate Program in Medical Sciences, Federal University of Fronteira Sul, Chapecó, SC Brazil

**Keywords:** Chronic spontaneous urticaria, Purinergic signaling, Treatment, Therapies

## Abstract

**Abstract:**

Urticaria is a skin lesion characterized by a central swelling area of varying sizes, usually surrounded by erythema and accompanied by itching or a burning sensation. Recurrence for at least six weeks characterizes chronic urticaria, a condition that has no cure, only treatment to minimize symptoms. The available pharmacological therapies show variable success and do not regulate the inflammatory balance of the skin in the long term. Therefore, it is essential to evaluate treatment possibilities to seek new symptom management approaches. In this regard, this study addresses the currently available therapies, linking the disease’s pathophysiology with purinergic signaling to identify new therapeutic possibilities. In this respect, extracellular ATP is related to mast cells degranulation in the skin, which affects urticaria. Considering other dermatological diseases that present an inflammatory response, the purinergic receptor P2X7 is related to inflammation in several pathological skin conditions, such as psoriasis, dermatitis and pruritus. P2X7 engagement also contributes to ATP-mediated mast cell degranulation. Therefore, evaluating the expression of purinergic receptors in the skin of patients with chronic spontaneous urticaria, along with ATP levels in urticarial lesions, may help elucidate the therapeutic potential of targeting this purinergic pathway.

**Key messages:**

- Many patients are refractory to licensed therapies of H1antihistamines and omalizumab.
- Cyclosporine and other immunosuppressants are studied for refractory patients.
- Probiotics as adjuncts to antihistamines are studied to reduce symptoms.
- P2X7 receptor links to inflammatory skin diseases and may offer new therapy approach.

**Graphical abstract:**

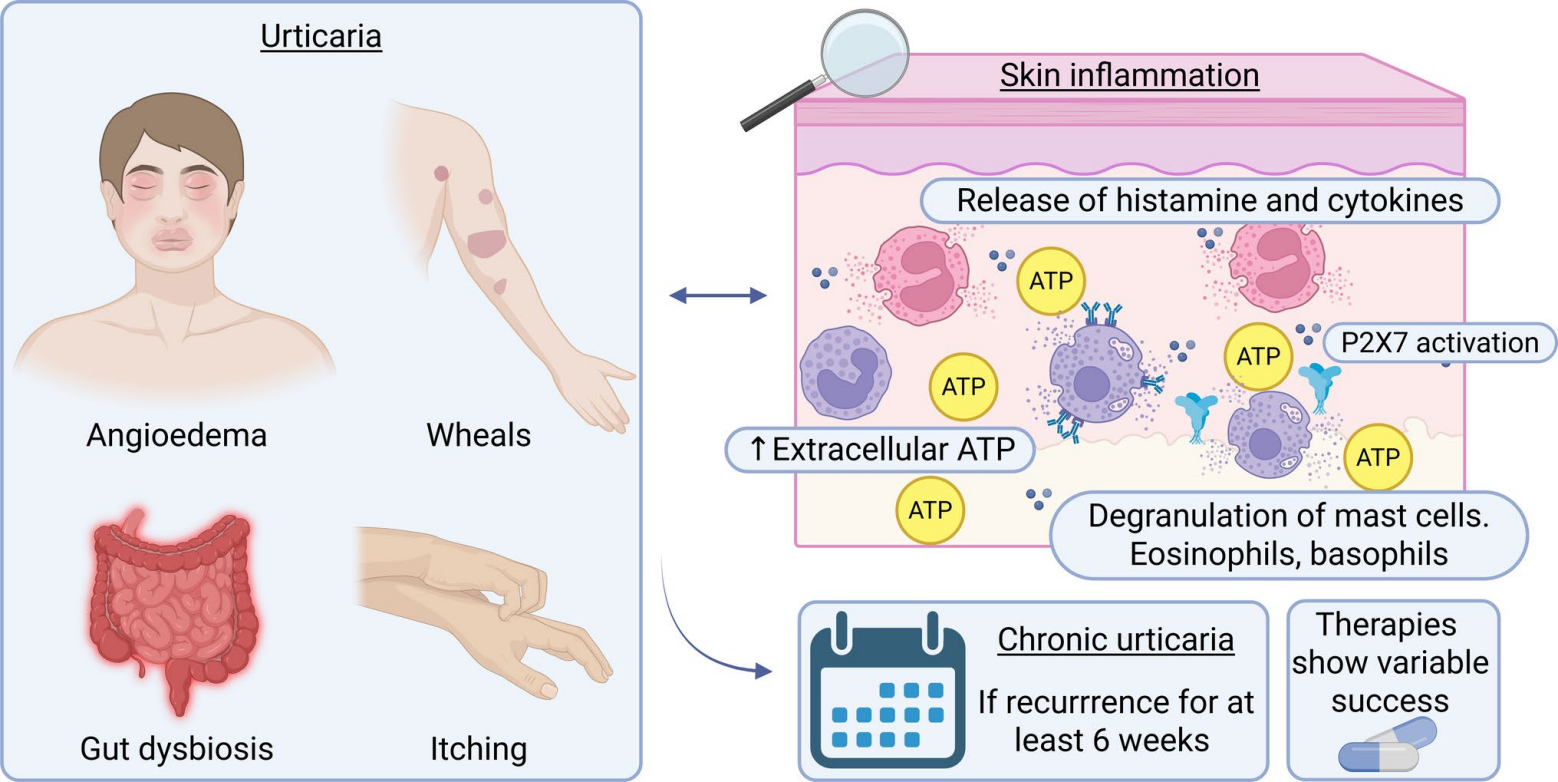

## Introduction

So far, chronic spontaneous urticaria (CSU) does not have a treatment that provides a cure, only symptom control [1]. Pharmacological therapy is often necessary, but its success varies. Furthermore, even when treatment reduces symptoms, there is no lasting adjustment of the skin’s inflammatory balance [1].

Therefore, considering that current treatments do not fully control symptoms in many patients with chronic urticaria, further investigations into effective and safe treatments for the disease are still needed [2]. Patients with autoimmune CSU, especially those with co-occurrence of IgE and IgG autoantibodies to the same target autoantigen, need new therapies that effectively control the disease [3]. In this regard, personalized approaches are being investigated based on endotypes and the identification of autoantibodies and other factors involved in pathogenesis [3].

In addition, extracellular signaling molecules such as ATP, purines, and pyrimidines related to regulating immune cell functions have become possible therapeutic focuses for various diseases, especially those related to mast cells [4]. Thus, this study aimed to address the treatments for CSU and its pathophysiology, linking it to its modulation by the purinergic signaling.

## Chronic urticaria

Urticaria is a type of skin condition defined by the development of wheals, angioedema, or both [5]. Wheals present a central swollen area of various sizes, usually with surrounding erythema, itching and burning sensation, and a transient period of lesion lasting up to 24 h followed by a return to the normal state of the skin without ecchymosis [5]. Angioedema manifests as deep, sudden, and pronounced swelling in the lower dermis and subcutaneous tissue or in the mucous membranes, with a sensation of tingling, burning, and pain lasting up to 72 h [5].

The condition is classified as acute or chronic based on how long symptoms last. Recurrent urticaria for at least six weeks defines chronic urticaria, while less than 6 weeks defines acute urticaria [5]. Regarding the stimulus that triggers urticaria, it can also be classified as spontaneous or inducible [5]. Spontaneous urticaria occurs without a direct relationship to a stimulus. In contrast, inducible urticaria can be triggered by environmental stimuli like cold, heat, pressure applied to the skin, physical exercise, water, vibration, and sunlight [5]. Furthermore, two endotypes of CSU are emerging: the autoimmune CSU (type IIb) mediated by IgG autoantibodies and the autoallergic CSU mediated by IgE autoantibodies (type I), both cases mediated by mast cell- and basophil-activating autoantibodies [3].

This chronic disease significantly impacts life quality, leading to reduced productivity, social interactions, and sleep quality, as well as an increase in psychological comorbidities [2, 6–10]. The incidence of mental disorders in patients with chronic urticaria is high, with depression and anxiety being the most prevalent [8]. Furthermore, if untreated, it can aggravate and affect the respiratory and gastrointestinal systems [11].

## Pathophysiology of urticaria

**Fig. 1:**
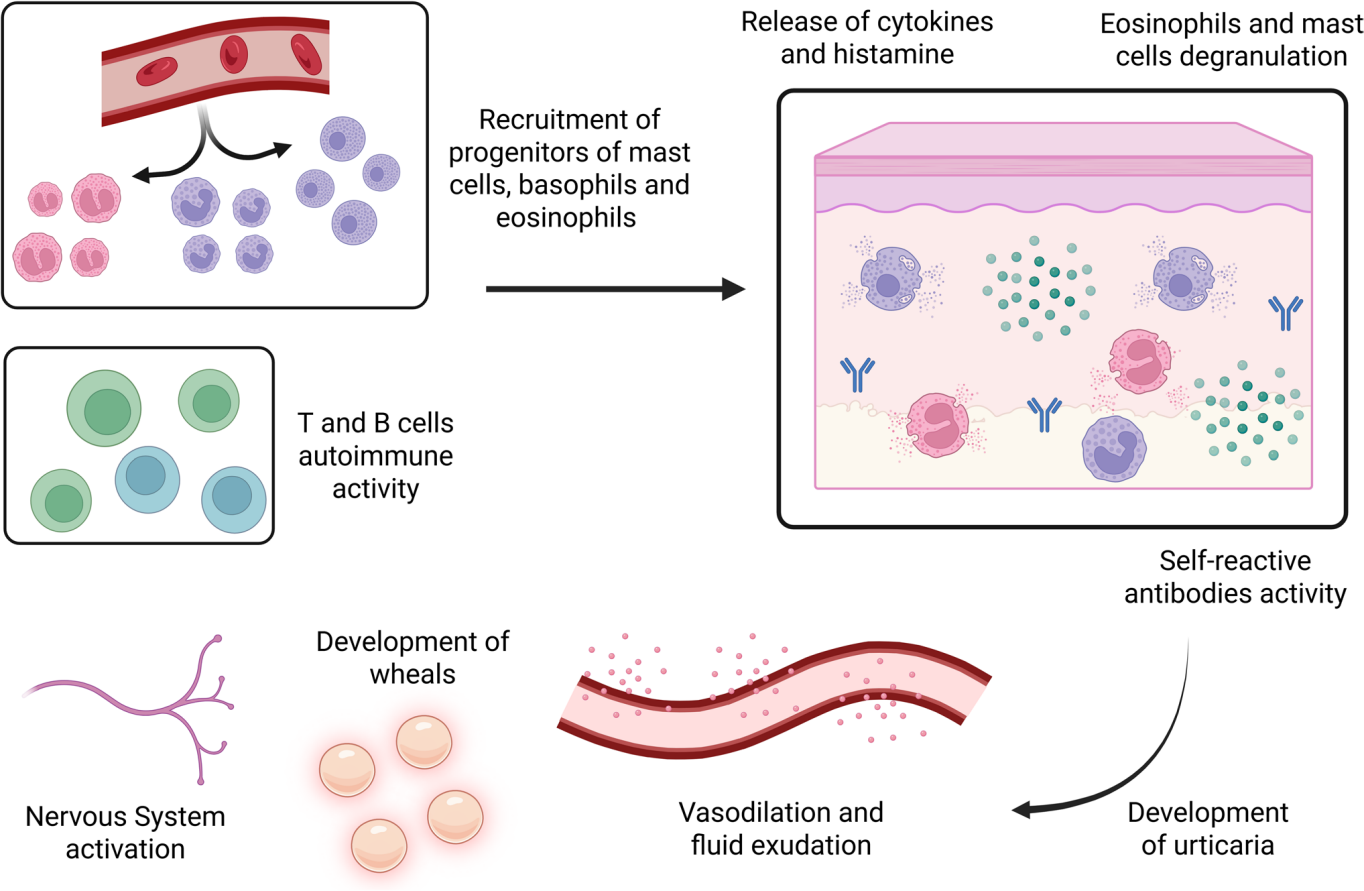
Pathophysiology of CSU. The activation of mast cells, eosinophils, and immune system cells stimulates the release of cytokines and histamines, which promotes vasodilatation, dermal inflammation, sensory nerve activation, and the development of urticaria lesions. Created with BioRender.com

Although the exact pathogenesis of inducible urticaria is unknown, this disorder probably results from the increased sensitivity of mast cells to environmental conditions. Similarly, the pathogenesis of CSU has not been fully elucidated, however, we present current hypotheses from the literature that may help explain its underlying mechanisms. The pathogenesis of CSU (Fig. 1) is directly related to the activation of mast cells, T cells, eosinophils, and other immune system cells [12].

CSU is predominantly caused by mast cell activation; however, emerging evidence suggests that eosinophils also affect symptom manifestation [13]. In this context, patients exhibit eosinopenia, a reduction in eosinophils in peripheral blood, due to their recruitment to the skin during active phases of the disease and their immunological destruction in the bloodstream [13].

The skin mast cell, particularly the tryptase and chymase positive subtype, is the primary effector cell in urticaria [14]. Activation of mast cells and basophils releases histamine and cytokines, triggering dermal inflammation [15]. The release of histamine, along with other mediators such as prostaglandin D2 (PGD2) and tumor necrosis factor (TNF) plays a role in the symptoms of CSU [16]. Thus, the symptoms of chronic urticaria are caused by mast cell activation and degranulation in the skin [17]. The signals of mast cell activation in urticaria are diverse and include cytokines and autoantibodies produced by self-reactive B cells and mediated by T cell–dependent mechanisms [5]. Elements released by mast cells, such as histamine, platelet-activating factor, cytokines and lipid mediators result in sensory nerve activation, vasodilation, plasma leakage, and recruitment of cells to urticarial lesions [5].

It is also believed that the pathogenesis of CSU involves an autoimmune component, that is, an autoimmune mechanism of activation of skin mast cells, which can be characterized by the presence of immunoglobulin E autoantibodies, IgE (type I or autoallergic), or immunoglobulin G autoantibodies, IgG (type IIb or autoimmune) [2, 18–20]. Both types involve FcεRI receptors and can occur simultaneously in the same patient [15]. Mast cells can be activated through the binding of these autoantibodies, specifically, IgG to FcεRI and IgE (i.e., IgG-antiFcεRI and IgG anti-IgE) and IgE to self-allergens (e.g., IgE-anti-thyroperoxidase and IgE-anti-IL-24) [3].

In this regard, signaling molecules such as Bruton’s tyrosine kinase (BTK) regulate FcεRI-mediated mast cell activation and B cell receptor-mediated antibody production [3]. Furthermore, other pathways may play a role in autoimmune CSU, including the JAK-STAT signaling pathway and the IL-17 and IL-23 axis [3]. A pilot study, including 45 participants (15 patients with atopic dermatitis, 15 patients with CSU, and 15 healthy controls) used blood samples to examine the serum levels of IL-5R using the enzyme-linked immunosorbent assay (ELISA) test [13]. They obtained a significant association between serum concentrations of interleukin-5 receptor (IL-5R) in patients with CSU compared to healthy individuals [13]. In lesional skin of CSU patients, an increase in IL-5-positive cells has been described and IL-5 is an important chemoattractant for eosinophils, eosinophils are significantly increased in CSU skin lesions [14].

Autoimmune CSU (type IIb) has an incidence of 8 to 40% of patients with CSU and is associated with high disease activity and autoimmune comorbidities, such as autoimmune thyroid disease [3]. Other autoimmune diseases are also commonly manifested in patients with CSU, such as pernicious anemia, vitiligo, celiac disease, type I diabetes, and rheumatoid arthritis [21]. In comparison, autoallergic CSU is present in more than 50% of patients with CSU [3].

In histological aspects, the wheals are characterized by edema of the upper and middle dermis with dilation and increased permeability of the upper dermis’s post-capillary venules and lymphatic vessels [5]. The wheals present a mixed inflammatory perivascular infiltrate of variable intensity, consisting of T cells, eosinophils, basophils, and other cells [5]. In non-lesional skin regions of patients with CSU, there is upregulation of adhesion molecules, eosinophil infiltration, altered cytokine expression, and, sometimes, an increase in the number of mast cells [5, 14].

## Treatments

Currently advised treatment options aim to target mast cell mediators, like histamine, or activators, like autoantibodies [5]. The general goal of treatment is to alleviate symptoms until spontaneous remission of urticaria occurs; to achieve it, pharmacological treatment should be continuous until it is no longer needed [5].

The international guideline developed jointly by the European Academy of Allergology and Clinical Immunology, the Global Allergy and Asthma European Network, the European Dermatology Forum, and the Asia Pacific Association of Allergy, Asthma and Clinical Immunology (EAACI/GA^2^LEN/EuroGuiDerm/APAAACI) from 2022 recommends that second-generation H1 antihistamines be the first-line therapy for chronic urticaria [5]. Patients who respond to treatment with a second-generation H1 antihistamine at a standard daily dosage may discontinue treatment after 3 to 6 months of uninterrupted complete control and total absence of signs and symptoms [22].

Medications such as loratadine, desloratadine, cetirizine, and levocetirizine can be used and are preferred over first-generation antihistamines because they have less penetration through the blood–brain barrier and cause fewer side effects [9, 19]. The most common side effects include fatigue, sedation, insomnia, and dyspepsia [23, 24].

Due to the frequent failure to obtain an adequate response with the licensed dosage, the second stage of treatment consists of increasing the medication dosage; however, this recommendation is based on expert opinions, without a clinical consensus being reached [25]. Therefore, in this antihistamine therapy, if there is no improvement in clinical symptoms, the dosage can be increased up to 4 times the standard dose in an off-label approach [5].

These antihistamines bind to and stabilize the inactive conformation of the histamine H1 receptor [1]. However, because histamine is not the only inflammatory mediator involved, this treatment is often insufficient to control the symptoms [1]. Approximately 61% of patients with chronic urticaria do not benefit from the standard dose and require additional antihistamine dosing [1]. In addition, it is estimated that 63.2% of patients refractory to the standard dose benefit from an increase in dosage 2 to 4 times [25]. Meanwhile, at least one-quarter of patients with chronic urticaria continue to have uncontrolled disease despite the increased dosage of second-generation antihistamines [3].

Regarding treatment with antihistamines, Xu and collaborators evaluated the combination of olopatadine hydrochloride with desloratadine citrate disodium in the treatment of urticaria. In this study with 114 patients with urticaria, it was observed that patients treated with the combination presented significant increases in the levels of IL-2 and IFN-γ and reductions in the levels of IL-4, number of wheals, size of wheals, and degree of itching [11]. With this, they demonstrated that the combination of antihistamines can also be an alternative to reduce the symptoms and the risk of recurrence of urticaria [11]. In addition, increased IL-2 levels were reported in a prospective study enrolling 59 antihistamine-resistant CSU patients treated with omalizumab [26]. After 16 weeks of treatment, responders showed significantly higher levels of IL-2, IL-5, IL-6, IL-22, and TNF-α compared with baseline [26].

Some inducible urticaria, such as dermographism, respond to antihistamines, while other forms, such as local heat urticaria, are typically resistant [27]. From 5 to 50% of patients with chronic urticaria do not achieve satisfactory disease control with antihistamine-based treatments [28]. Thus, immunomodulators may also be an integral part of the treatment for chronic urticaria [10]. Moreover, adjuvant therapies are primarily indicated for patients who do not respond to treatment and the increased doses of antihistamines [29]. Therefore, other treatments can be applied in the therapy of chronic urticaria when the disease is not controlled by antihistamines (Table 1).

**Table 1:** Main therapeutic possibilities

Therapy	Stage of Treatment	Indication	Action	Adverse effects	Reference
Second-generation H1 antihistamines	1 st line in EAACI/GA2LEN/EuroGuiDerm/APAAACI guideline	Start with standard dose and if needed increase the dose up to 4 times	Stabilization of the inactive conformation of the histamine H1 receptor	Fatigue, sedation, drowsiness, and dyspepsia	[1, 5, 23, 24]
Omalizumab	2nd line in EAACI/GA2LEN/EuroGuiDerm/APAAACI guideline	When refractory, add on omalizumab to antihistamines	Immunoglobulin E blockade	Anaphylaxis, nasopharyngitis, dermatitis, upper respiratory tract infection, and headache	[5, 10, 19]
Remibrutinib	-	CSU in adult patients who remain symptomatic despite H1 antihistamine treatment	Highly selective BTK inhibitor that blocks BTK-mediated degranulation of mast cells and basophils downstream of FcεRI	Nasopharyngitis, bleeding, headache, nausea and abdominal pain	[30, 31]
Cyclosporine	3rd line in EAACI/GA2LEN/EuroGuiDerm/APAAACI guideline	Off-label for urticaria, indicated only for patients with severe disease refractory to any dose of antihistamine and Omalizumab. Use in combination with antihistamine	Immunosuppression	Kidney dysfunction, hypertension, nausea, vomiting and headaches	[5, 9, 19]

### Omalizumab

The 2022 EAACI/GA^2^LEN/EuroGuiDerm/APAAACI guideline lists omalizumab as the only other licensed treatment for urticaria for use in patients who do not show improvement with antihistamine treatment [5]. Omalizumab is a recombinant humanized monoclonal antibody that blocks immunoglobulin E, known as IgE, and this drug is indicated for treating CSU refractory to antihistamines [19]. It acts by binding to free IgE and inhibiting interaction with the FceRI receptor on basophils and mast cells, thus preventing their activation and also decreasing free IgE levels, leading to the downregulation of FceRI receptors [9].

The response to treatment may depend on the disease endotype; patients with CSU type I who have IgE autoantibodies against allergens such as thyroid peroxidase (TPO) generally respond effectively to omalizumab [19]. On the other hand, patients with type IIb are less likely to respond to omalizumab, and the onset of the response to the medication is slower [19]. Additionally, this medication can cause adverse effects such as pharyngeal edema, nasopharyngitis, eczema, upper respiratory tract infection, and headache [10]. Very rarely, omalizumab injections can induce severe reactions, including anaphylaxis [3].

In an analysis of treatment with omalizumab, Chen and colleagues (2024) observed that patients who show insufficient response to the medication after four weeks of treatment may benefit from continuing the treatment for 16 weeks [20]. However, even then, the response to treatment may not be complete or sufficient, with a response rate of 71.9% observed at the end of the 16-week treatment period [20]. The early response, defined as the resolution of CSU symptoms within 4 weeks from the initiation of omalizumab treatment had a response rate of 28,05% (23 early responders/82 patients) [20]. In patients who achieve inadequate disease control with the approved omalizumab regimen of 300 mg every four weeks, dose escalation strategies may be considered, including higher doses, reduced dosing intervals, or both [5]. Evidence supports the use of omalizumab at doses up to 600 mg administered every two weeks in individuals with suboptimal response to standard dosing. Although such adjustments constitute off-label use, this should be clearly communicated to patients [5].

### Immunosuppressants

Immunosuppressive medications may help reduce autoantibodies and activated T cells, contributing to the treatment of chronic urticaria [5]. However, due to the side effects of these medications, their use requires active monitoring of adverse effects [10]. Chronic autoimmune spontaneous urticaria (IIb) is associated with a poor response to second-generation antihistamines and omalizumab while presenting a good response to cyclosporine [3].

Cyclosporine is an immunosuppressive drug used as a complement in combination with antihistamine or omalizumab [9]. The mechanism of this drug involves inhibition of cell mediated immunity by downregulating Th1 lymphocyte responses and T cell-dependent antibody formation by B lymphocytes [32]. It also has inhibitory effects on anti-IgE-induced histamine release from human basophils and skin mast cells in vitro [32]. Its use is associated with side effects such as renal dysfunction and hypertension, which is why this therapy is only applied as a complementary off-label therapy in patients with severe CSU who do not respond to treatment with antihistamines and omalizumab [9, 19]. In such cases, the 2022 EAACI/GA^2^LEN/EuroGuiDerm/APAAACI guideline recommends cyclosporine doses of 3.5–5 mg/kg per day for patients with urticaria [5]. A study involving thirty patients with severe chronic idiopathic urticaria occurring daily or almost daily for more than six weeks, with poor response to antihistamine therapy, evaluated the efficacy of cyclosporine [32]. Patients were randomized to receive cyclosporine at a dose of 4 mg/kg daily (Sandimmun, n = 20) or placebo (n = 10), the overall response rate to cyclosporin was 65% until eight weeks [32].

Due to the different symptom responses of chronic urticaria, other immunosuppressants are also presented as off-label treatments, such as methotrexate, azathioprine, and mycophenolate mofetil [10, 19]. In addition, dupilumab, a monoclonal antibody targeting the IL-4 receptor α chain (IL-4Rα) and thereby inhibiting IL-4 and IL-13 signaling, is currently under investigation for CSU, given the increased expression and serum levels of IL-4 and IL-13 observed in lesional skin, blood, or both [3]. Beyond that, a phase 1 study of mepolizumab in chronic spontaneous urticaria is ongoing (NCT03494881), mepolizumab neutralizes IL-5, leading to reduced eosinophil survival and activity. [3].

### Remibrutinib

The U.S. Food and Drug Administration (FDA) recently approved remibrutinib, a kinase inhibitor indicated for the treatment of CSU in adult patients who remain symptomatic despite H1 antihistamine treatment [30]. BTK is a cytoplasmic kinase belonging to the TEC (tyrosine kinase expressed in hepatocellular carcinoma) kinase family. In CSU, activation of BTK downstream of the high-affinity IgE receptor (FcεRI) in mast cells and basophils leads to the release of histamine and other proinflammatory mediators responsible for disease manifestations [31]. In addition, activation of BTK is responsible for autoantibody production by B cells in chronic spontaneous urticaria. Remibrutinib is an oral, highly selective BTK inhibitor that blocks BTK-mediated degranulation of mast cells and basophils downstream of FcεRI, which prevents the release of histamine and other proinflammatory mediators that lead to itch, hives, angioedema, or a combination of these symptoms [31].

The main side effects of remibrutinib are nasopharyngitis, bleeding, headache, nausea and abdominal pain [30]. Regarding the risk of bleeding it is indicated monitoring for signs, the interruption if treatment if bleeding is observed and the interruption 3 to 7 days pre- and post-surgery or invasive procedures, beyond that, the use of antithrombotic agents concomitantly may increase the risk of bleeding [30].

A randomized, double-blind, placebo-controlled, phase 2b trial evaluated remibrutinib during 12 weeks in patients inadequately controlled with second-generation H1-antihistamines, with at least moderately active CSU [33]. Overall, 311 patients were randomized and it was evaluated the weekly Urticaria Activity Score (UAS7) which integrates weekly assessments of pruritus and wheal severity, yielding a total score ranging from 0 to 42, with higher values reflecting increased disease activity. Clinical improvement in hives and itching was evident from week 1 and maintained throughout the 12 week treatment period. In the 25 mg twice daily dose, as many as 41.9% of patients achieved UAS7 = 0, and 55.8% achieved UAS7 ≤ 6 at week 12 compared to 14.3% and 28.6% of patients in the placebo arm, respectively [33].

### Probiotics adjuvant therapies

This section addressing adjuvant therapies, such as probiotics, is intended to provide a comprehensive overview of therapeutic approaches that have been explored in CSU, beyond those currently recommended in clinical guidelines. Although probiotics are not part of standard treatment algorithms, experimental and clinical studies have reported potential symptomatic benefits in selected patient populations.

Gut dysbiosis accompanies CSU [34]. Shi et al. (2023) [35] conducted a bidirectional two-sample mendelian randomization study and confirmed the bidirectional potential causal relationship between urticaria and gut microbiota. The changes in the gut microbiome in CSU might decrease short-chain fatty acids, increase lipopolysaccharide levels, and promote skin inflammation induced by mast cells [34]. In addition, Luo et al. (2023) [36] also signaled that alterations in gut microbiota and metabolites might contribute to immune and inflammatory pathways involved in the pathogenesis of CSU.

The microbiome of CSU patients is characterized by reduced diversity and markedly lower levels of short chain fatty acids-producing gut bacteria, in addition, low blood levels of caproate and low levels of short chain fatty acids-producing bacteria were linked to increased levels of opportunistic pathogens such as *Klebsiella pneumonia* and *Escherichia coli* [34]. Zhu and colaborators (2024) observed the microbiome of CSU increases mast cell-driven skin inflammation, intestinal permeability, and blood lipopolysaccharide level [34]. Additionally, *Roseburia hominis* and short-chain fatty acid attenuate mast cell-driven skin inflammation, this bacterium was negatively correlated with blood lipopolysaccharide levels of CSU patients [34]. Furthermore, *Roseburia hominis* markedly increased gut short chain fatty acids levels and protected from skin mast cell degranulation in adoptively transferred mice. Whereas *Klebsiella pneumoniae* and lipopolysaccharide exacerbate mast cell-driven skin inflammation [34].

The use of probiotics emerges as promising adjuvant therapy for the disease. This is attributed to alterations in the gut microbiota of patients with chronic urticaria, which could be corrected through probiotic supplementation [37]. Furthermore, Th-2 cells (type 2 helper T lymphocytes) play a critical role in the pathogenesis of allergic reactions and in producing urticaria-related antibodies [28]. Probiotics can prevent an allergic response due to their anti-inflammatory effects, shifting the Th1/Th2 balance towards Th1 by inhibiting Th2 cytokines or indirectly inducing the production of IL-10 and regulatory T cells [37]. These probiotics stimulate the synthesis of anti-inflammatory mediators, promoting an anti-inflammatory environment that protects against chronic urticaria [16].

Atefi et al. (2022) [28] evaluated the efficacy and safety of LactoCare (*Lactobacillus rhamnosus, Lactobacillus casei, Lactobacillus acidophilus, Bifidobacterium breve, Lactobacillus bulgaricus, Bifidobacterium longum, Streptococcus thermophilus*), a synbiotic compound (prebiotic and probiotic), in the treatment of chronic urticaria. Their analysis was structured through a randomized clinical study with 42 patients (21 in the antihistamine control group and 21 in the antihistamine + probiotic intervention group) with CSU for 8 weeks. The researchers observed that in patients who remained symptomatic despite antihistamine treatment, the combination therapy may reduce itching and total score measured by UAS7, in addition to improving life quality measured by the Dermatology Life Quality Index (DLQI) [28]. Thus, the LactoCare compound appeared as a potential adjuvant therapy for chronic urticaria.

Bi et al. (2021) [37] evaluated the probiotic formula Yimingjia® in the adjunctive treatment of chronic urticaria in children. In this study, 206 children diagnosed with chronic urticaria were randomly divided into a treatment group (104) and a placebo group (102). Children in both groups were treated with desloratadine dry suspension, and the ones in the treatment group additionally received Yimingjia®. Their study demonstrated that combining probiotics accompanied by long-acting antihistamine medication for four weeks can reduce clinical symptoms, wheals, size, and frequency of manifestations in children compared to those receiving only medication [37]. This study corroborates the results of Atefi et al. (2022) [28], which show that the combination of probiotics is beneficial as an adjuvant therapy for chronic urticaria. Thus, other probiotic adjuvant therapies can be investigated to minimize disease symptoms.

### Perspective for future treatments

As described before, the management of symptoms is frequently a challenge regarding the difficulty in the response of current treatments. At least one-quarter of patients with chronic urticaria continue to have uncontrolled disease despite the increased dosage of second-generation antihistamines, the first line treatment [3], and even the combination of immunosuppressants treatments might show insufficient response [20]. Even the use of adjuvant therapies, such as probiotics, still needs to be standardized. Although probiotics have been investigated experimentally in the treatment of urticaria, their use is not currently recommended in clinical guidelines.

Considering the difficulties encountered in controlling the manifestations of CSU with the current therapies, the investigation of mechanisms involved in the disease manifestations could provide new approaches for the treatment. In this scenario, the purinergic signaling investigation might bring novel possibilities. This emerges from the link between purinergic signaling and the pathology of various skin conditions. Both the purinergic receptors expression and the ATP levels in CSU lesions could be investigated to elucidate the potential of acting over those receptors, stimulating them or even inhibiting them to obtain a controlled response of the disease.

## Purinergic signaling and urticaria

Burnstock (1972) described the purinergic signaling hypothesis and demonstrated that ATP, beyond an intracellular energy source, is also involved in extracellular signaling [38, 39]. In this context, purines and pyrimidines play key roles in cellular signaling processes such as proliferation, differentiation, and death, as well as in neurotransmission, neuromodulation, secretion, chemoattraction, and platelet aggregation [39–41]. This signaling pathway involves adenosine receptors, known as P1 receptors, which include four subtypes of G protein-coupled receptors (A1, A2A, A2B, and A3), and for ATP, ADP, and UTP, called P2 receptors [42].

In literature, the relationship between purinergic system modulation and the management of urticaria is scarce, but here is highlighted evidence suggesting that targeting the purinergic signaling could serve as a potential strategy for developing new treatments for the disease. This motivation arises from the involvement of purinergic signaling in skin conditions such as inflammation, wound healing, pain, psoriasis, scleroderma, warts, and cancer [43]. Although there is evidence supporting the involvement of P2X7 receptors in skin conditions, which are following addressed, the direct mechanistic link between purinergic receptors and the pathophysiology of CSU still needs to be investigated. To the best of our knowledge, to date there are no studies that have examined P2X7 expression in the skin of patients with CSU or ATP levels in urticarial lesions. This represents a gap that should be addressed in future research.

**Fig. 2:**
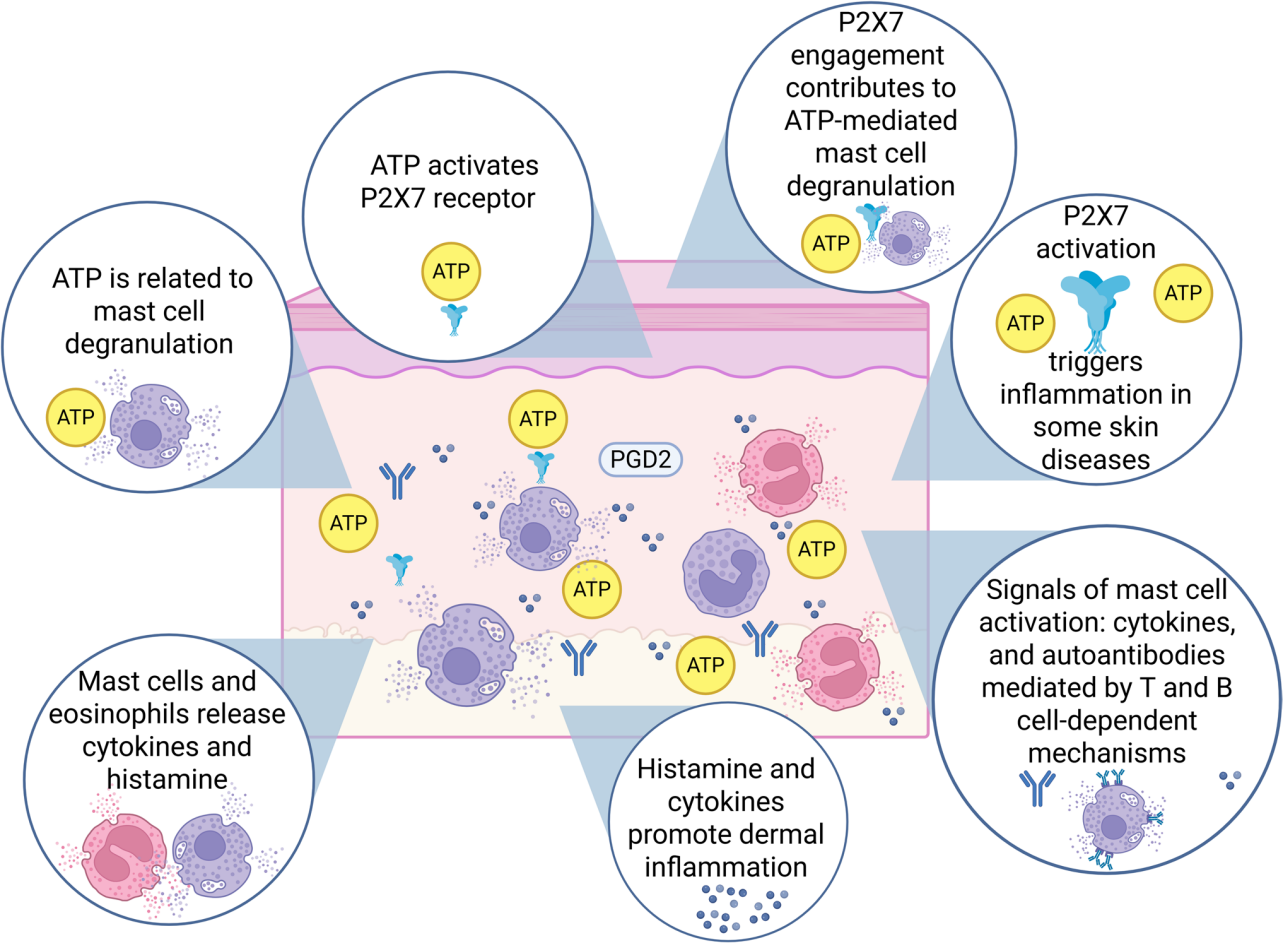
Purinergic signaling and skin inflammation in CSU. Extracellular ATP is related to the degranulation of mast cells in the skin, mast cells affects urticaria by the release of histamines and cytokines, triggering dermal inflammation. The signals of mast cell activation are diverse and include cytokines and autoantibodies produced by self-reactive B cells and mediated by T cell-dependent mechanisms. The purinergic receptor P2X7 is related to inflammation in several pathological skin conditions, such as psoriasis and dermatitis. This purinergic receptor is activated by extracellular ATP, intensifying the release of inflammatory mediators that trigger skin lesions. Created with BioRender.com

Under pathological conditions like inflammation, hypoxia, injury, and stress, the ATP component of extracellular cotransmission becomes elevated [39, 42]. While in normal physiological states, the levels of ATP and adenosine are kept low through the regulation of enzymes and transporters [42]. ATP activates P2 purinergic receptors on the cell membrane, triggering signaling pathways that produce inflammatory responses [44] (Fig. 2). Mast cell degranulation in the skin occurs due to ATP released during axonal reflexes at sensory nerve endings, which also leads to the dilation of skin blood vessels [43, 45, 46]. P2X7 is one of the main receptor driving ATP-induced mast cell degranulation in humans [47]. This mast cell degranulation affects various conditions, such as urticaria [48]. Thus, ATP plays a role in the inflammatory process by triggering the release of histamine from mast cells, the production of prostaglandins, and the production and release of cytokines from immune system cells [49]. These findings support the potential for therapeutic evaluation involving the modulation of ATP receptors in patients with CSU.

Table 2 shows details of P2 receptors. Despite the weak effect of P2X4 receptor-mediated in mast cells, this receptor can enhance mast cells responses induced by different stimuli, including Ag-induced IgE-FcεRI activation, Gi-coupled EP_3_ and A3 receptor activation [4]. The release of ATP from skin cells can occur through mechanical stimuli, such as scratching, which may lead to the activation of mast cells by ATP via the P2X4 receptor, thereby intensifying allergic reactions [4]. On the other hand, ATP released by damaged cells and in response to mechanical disturbances is involved in tissue healing and repair [43]. P2Y2 receptors play a role in epidermal homeostasis [50], while P2Y1 and P2X7 receptors play a role in skin inflammation [43].

**Table 2:** P2 receptors effect in skin

Receptor	Ligand	Effect	Reference
P2X4	ATP	The P2X4 receptor-mediated effect is weak in mast cells. However, it enhanced the mast cells responses induced by different stimuli, including Ag-induced IgE-FcεRI activation, Gi-coupled EP_3_ and A3 receptor activation	[4]
P2X7	ATP	Mast cells degranulation. They are expressed in langerhans cells, keratinocytes, skin fibroblasts and in those cells it enhances Th17-mediated immune responses, contributing to psoriasis-related inflammation and pruritus	[43, 45–47, 51]
P2Y2	ATP	They are expressed by human keratinocytes and play a major role in epidermal homeostasis, ATP, UTP and ATPgS were found to stimulate the proliferation of keratinocytes and under static conditions cultured human keratinocytes release ATP	[50]

In this regard, the purine receptor P2X7 plays a central role in the development of inflammatory responses, and it is found in several cells of the immune system, acting in the release of a large part of the pro-inflammatory cytokines [52]. From this perspective, studies indicate an increased regulation of P2X7 activities in lesions of patients with psoriasis; this purinoceptor is found in the keratinized epithelium and can, therefore, trigger inflammation directly at the site where psoriasis plaques form [52]. Furthermore, it is highlighted that allergic contact dermatitis can be prevented by interfering with P2X7 receptor signaling since this receptor plays a role in the extracellular release of ATP in the skin [43].

Salcman et al. (2024) used primary blood-derived human mast cells as a model and obtained the contribution of the receptors involved in mast cell degranulation. They observed human primary mast cells degranulate upon ATP but not ADP stimulation, beyond that, the presence of the P2X7 inhibitor, A438079, led to a decrease in mast cells degranulation, while the use of the P2X1 (NF449) and P2X4 (5BDBD) inhibitors, alone or in combination, had no effect [47]. However, the combination of the P2X1 and P2X7 inhibitors resulted in the complete inhibition of mast cells degranulation, suggesting that the P2X1 and P2X4 receptors exerted efflux and influx influences on P2X7 receptor activation, irrespective of the IL-33 priming concentration used [47]. These findings reinforce the role of P2X7 as the principal receptor driving ATP-induced degranulation in humans.

Thus, considering the inflammatory role that activation of the P2X7 receptor triggers in patients with psoriasis and allergic dermatitis and the release of inflammatory mediators that trigger skin lesions, it is suggested that, similarly, some regulation of the inflammation observed in chronic urticaria can be obtained through modulation of this receptor. From this perspective, modulation of the purinergic receptor P2X7 represents a promising strategy for improving the treatment of CSU. This means that exploring similar approaches to those suggested to be evaluated in psoriasis, such as P2X7 receptor antagonism [52], could reduce the inflammatory response and relieve skin symptoms, and should be further investigated in CSU.

Regarding pruritus, evidence indicates ATP acting on P2 receptors in skin sensory nerves participates in pruritus, which can emerge from multiple skin conditions, such as psoriasis and chronic urticaria [51]. In addition, authors showed keratinocytes, fibroblasts, and primary sensory nerves express P2 receptors which are involved in pruritus, although the P2 purinergic signaling mechanism in itch remains incompletely understood and needs further investigation [51]. P2X7 receptors are expressed in langerhans cells, keratinocytes, skin fibroblasts and in those cells it enhances Th17-mediated immune responses, contributing to psoriasis-related inflammation and pruritus [51]. Activation of P2 purinergic receptors induces pruritic behavior, while their inhibition alleviates pathological skin pruritus. Beyond that, P2X7 receptor was studied in systemic sclerosis, a connective tissue disorder presenting fibrosis of the skin and internal organs. Gentile et al. (2017) isolated fibroblasts by skin biopsy from 9 systemic sclerosis patients and 8 healthy controls. They observed P2X7 receptor expression and Ca^2+^ permeability induced by the selective P2X7 receptor agonist 2′−3′-O-(4-benzoylbenzoyl) ATP (BzATP) were markedly higher in systemic sclerosis than control fibroblasts [53]. This demonstrates that the P2X7 receptor in skin fibroblasts can be used as a target for skin diseases.

Inflammation in the skin is also related to the action of adenosine, which acts through four G protein-coupled receptors (A1, A2A, A2B, and A3) [42]. Thus, considering the regulatory effects of these receptors on immune signaling pathways, their modulation may be a strategy for anti-inflammatory intervention in skin diseases [42]. Adenosine acts as an endogenous regulator of inflammatory processes in the skin, facilitating inflammation to promote healing [42]. Thus, manipulating adenosine receptor functions in inflammatory skin diseases may play pathological and therapeutic roles [42].

Previous studies have shown that human keratinocytes predominantly express A2B adenosine receptors, while A1, A2A, and A3 receptors are not consistently detected [42]. Although the presence of other adenosine receptor subtypes has been suggested, the available evidence remains inconsistent, and no definitive expression pattern has been established. The effects of adenosine on epidermal cell proliferation remain inconsistent in the literature, likely reflecting variations in the expression profiles and combinations of adenosine receptor subtypes [42]. Adenosine has been reported to promote proliferative responses in keratinocytes and melanocytes through A2A receptor activation, whereas stimulation of A3 receptors has been associated with growth arrest. Moreover, A2B receptors have been implicated in both proliferative and antiproliferative effects, underscoring the complex and sometimes opposing roles of adenosine signaling in keratinocyte biology [42].

Additionally, during wound healing, adenosine modulates fibroblast activity with opposing effects on collagen and extracellular matrix production. Activation of A2A receptors promotes collagen synthesis in dermal fibroblasts, partly through the induction of mediators such as interleukin-13 and connective tissue growth factor, whereas A2B receptor signaling has been associated with inhibition of collagen production [42]. Thus, adenosine may act as either a pro- or anti-inflammatory mediator, depending on the pattern of receptor expression in keratinocytes.

Furthermore, the association between serum concentration of interleukin-5 receptor (IL-5R) and the increased expression and serum levels of IL-4 and IL-13 observed in lesional skin, blood, or both in patients with CSU also suggests that therapies targeting them may be evaluated [3, 13]. This also demonstrates other possible approaches to the therapeutic evaluation of CSU.

## Conclusions and future perspectives

Currently, available treatments for CSU involve the use of second-generation antihistamines and immunomodulation with omalizumab. However, the difficulty in controlling symptoms in many patients highlights the need to investigate new therapeutic approaches. In this regard, adjuvant therapies with probiotics are being studied to complement treatment. Furthermore, the association between extracellular ATP and the skin’s inflammatory response reveals possible relationships between the manifestation of urticaria symptoms and the purinergic system. Thus, it is suggested future studies evaluate the P2X7 expression in the skin of patients with CSU and ATP levels in urticarial lesions to evaluate new therapeutic possibilities for patients.

## Data Availability

All data is available online.
